# Outcomes and Mechanisms Associated With Selective Thalamic Neuronal Loss in Chronic Traumatic Brain Injury

**DOI:** 10.1001/jamanetworkopen.2024.26141

**Published:** 2024-08-06

**Authors:** Rebecca E. Woodrow, Julia Grossac, Young T. Hong, Stefan Winzeck, Thomas Geeraerts, Sudhin A. Shah, Alexander R. D. Peattie, Anne E. Manktelow, Joanne G. Outtrim, Nicolas A. Karakatsanis, Nicholas D. Schiff, Tim D. Fryer, David K. Menon, Jonathan P. Coles, Emmanuel A. Stamatakis

**Affiliations:** 1 University Division of Anaesthesia, University of Cambridge, Addenbrooke’s Hospital, Cambridge, United Kingdom; 2 Department of Clinical Neurosciences, University of Cambridge, Cambridge Biomedical Campus, Cambridge, United Kingdom; 3 Wolfson Brain Imaging Centre, University of Cambridge, Cambridge Biomedical Campus, Cambridge, United Kingdom; 4 BioMedIA Group, Department of Computing, Imperial College, London, United Kingdom; 5 Department of Radiology, Weill Cornell Medicine, New York, New York; 6 Department of Neurology, Brain and Mind Research Institute, Weill Cornell Medicine, New York, New York

## Abstract

**Importance:**

The chronic neuronal burden of traumatic brain injury (TBI) is not fully characterized by routine imaging, limiting understanding of the role of neuronal substrates in adverse outcomes.

**Objective:**

To determine whether tissues that appear healthy on routine imaging can be investigated for selective neuronal loss using [^11^C]flumazenil (FMZ) positron emission tomography (PET) and to examine whether this neuronal loss is associated with long-term outcomes.

**Design, Setting, and Participants:**

In this cross-sectional study, data were collected prospectively from 2 centers (University of Cambridge in the UK and Weill Cornell Medicine in the US) between September 1, 2004, and May 31, 2021. Patients with TBI (>6 months postinjury) were compared with healthy control participants (all aged >18 years). Individuals with neurological disease, benzodiazepine use, or contraindication to magnetic resonance imaging were excluded. Data were retrospectively collated with nonconsecutive recruitment, owing to convenience and scanner or PET ligand availability. Data were analyzed between February 1 and September 30, 2023.

**Exposure:**

Flumazenil voxelwise binding potential relative to nondisplaceable binding potential (BP_ND_).

**Main Outcomes and Measures:**

Selective neuronal loss identified with FMZ PET was compared between groups on voxelwise and regional scales, and its association with functional, cognitive, and psychological outcomes was examined using Glasgow Outcome Scale (GOS) scores, measures of sustained executive attention (animal and sustained fluency), and 36-Item Short Form Health Survey (SF-36) scores. Diffusion tensor imaging was used to assess structural connectivity of regions of cortical damage, and its association with thalamic selective neuronal loss.

**Results:**

In this study, 24 patients with chronic TBI (mean [SD] age, 39.2 [12.3] years; 18 men [75.0%]) and 33 healthy control participants (mean [SD] age, 47.6 [20.5] years; 23 men [69.7%]) underwent FMZ PET. Patients with TBI had a median time of 29 (range, 7-95) months from injury to scan. They displayed selective neuronal loss in thalamic nuclei, over and above gross volume loss in the left thalamus, and bilateral central, mediodorsal, ventral-lateral dorsal, anterior, and ventral anterior thalamic nuclei, across a wide range of injury severities. Neuronal loss was associated with worse functional outcome using GOS scores (left thalamus, left ventral anterior, and bilateral central, mediodorsal, and anterior nuclei), worse cognitive outcome on measures of sustained executive attention (left thalamus, bilateral central, and right mediodorsal nuclei), and worse emotional outcome using SF-36 scores (right central thalamic nucleus). Chronic thalamic neuronal loss partially mirrored the location of primary cortical contusions, which may indicate secondary injury mechanisms of transneuronal degeneration.

**Conclusions and Relevance:**

The findings of this study suggest that selective thalamic vulnerability may have chronic neuronal consequences with relevance to long-term outcome, suggesting the evolving and potentially lifelong thalamic neuronal consequences of TBI. FMZ PET is a more sensitive marker of the burden of neuronal injury than routine imaging; therefore, it could inform outcome prognostication and may lead to the development of individualized precision medicine approaches.

## Key Points

**Question:**

Can [^11^C]flumazenil (FMZ) positron emission tomography (PET) be used to identify selective neuronal loss associated with poor functional outcome after chronic traumatic brain injury (TBI)?

**Findings:**

This cross-sectional study compared 24 patients with chronic TBI with 33 healthy control participants using FMZ PET. Patients with chronic TBI displayed selective neuronal loss in specific thalamic nuclei, which was associated with worse long-term functional outcome, mirroring regions of cortical contusion suggestive of transneuronal degeneration.

**Meaning:**

These findings suggest that selective thalamic vulnerability may have chronic neuronal consequences with relevance to long-term outcome.

## Teaser

This cross-sectional study assesses the ability of [^11^C]flumazenil positron emission tomography (FMZ PET) to detect selective neuronal loss after chronic traumatic brain injury and examines the association between neuronal loss and long-term functional, cognitive, and psychological outcomes.

## Introduction

Poor cognitive and functional outcomes after traumatic brain injury (TBI) remain a major concern for public health. Understanding the pathophysiologic processes that result in poor functional outcome is essential.^1^ Radiographic computed tomography and magnetic resonance imaging (MRI) are commonly used to identify evidence of structural brain injury, such as focal contusions, hemorrhage, and traumatic axonal injury.^2^ However, these modalities lack insight into the neuronal integrity of tissue that appears healthy and its association with outcome. Improved understanding of diffuse injury and selective neuronal loss is important for assessing the entire burden of injury, for developing novel neuroprotective strategies, and for estimating eventual clinical and neuropsychological outcome.

Positron emission tomography (PET) imaging of the radioligand [^11^C]flumazenil (FMZ), which binds to the central benzodiazepine receptor, can be used as a marker of selective neuronal loss, whereby individual neuronal death remains supported by viable extracellular matrix and tissue bulk, as distinct from pan necrosis seen in MRI where there is complete cellular loss.^3^ In chronic TBI, investigators have found widespread reductions in FMZ binding potential (marker of selective neuronal loss) within bilateral frontal, temporal, and thalamic regions, which correlated with reduced intelligence^4^ and persistent cognitive problems,^5,6^ despite no visible structural damage on MRI. Longitudinal analyses reported broad FMZ binding potential decreases in subacute TBI that persisted chronically in frontal cortices and thalamic regions,^7^ whereby increases toward healthy levels in these regions correlated with improvement in executive attention. These findings highlight the utility of FMZ PET for understanding the burden of neuronal injury and estimating functional outcome.

The aforementioned studies demonstrated the increased sensitivity of FMZ PET to the burden of neuronal injury and its behavioral relevance in small sample sizes (n = 5-11), predominantly centered on the thalamus and frontal regions. This sensitivity requires further exploration in a larger sample with a greater breadth of long-term outcome measures, to improve understanding of the burden of neuronal injury seen in chronic TBI that may not present on routine imaging.

In this study, we investigated 2 hypotheses: (1) FMZ PET can be used to demonstrate chronic selective neuronal loss (>6 months postinjury) within brain regions that appear structurally healthy, and (2) such loss is particularly centered on the thalamus, with an association with long-term outcome. We further explored whether changes in thalamic integrity were associated with thalamic connections to cortical regions that had sustained damage, potentially driven by secondary injury mechanisms. Collectively, our aim was to substantiate the ability of FMZ PET to characterize the long-term burden of neuronal injury after TBI and to increase our understanding of long-term thalamic injury.

## Methods

### Participants

This cross-sectional study enrolled participants during the acute phase after TBI, with data collected prospectively from 2 centers: University of Cambridge in Cambridge, UK (n = 19), and Weill Cornell Medicine (WCM; n = 5) in New York, NY. For the Cambridge cohort, ethical approval was obtained from the Cambridgeshire Research Ethics Committee, and written informed consent (or written assent from next of kin where appropriate) was obtained from all participants. For the WCM cohort, study participants were recruited after written consent was obtained in accordance with approval granted by the WCM Institutional Review Board. The study followed the Strengthening the Reporting of Observational Studies in Epidemiology (STROBE) guideline.

All patients experienced a TBI severe enough to warrant acute neuroimaging that identified evidence of traumatic injury. The data reported were collected between September 1, 2004, and May 31, 2021, and were retrospectively collated with nonconsecutive recruitment to a follow-up imaging protocol targeting the chronic phase postinjury using MRI and FMZ PET, owing to convenience and scanner or PET ligand availability. At this chronic time point, patients with TBI were at least 6 months postinjury and were compared with 33 healthy control participants (n = 16 and 17 in the Cambridge and WCM cohorts) who underwent an identical imaging protocol. All participants were aged older than 18 years. Individuals with other neurological disease, benzodiazepine use, or contraindication to MRI were excluded. Data were analyzed between February 1 and September 30, 2023.

### Image Acquisition and Processing

[^11^C]Flumazenil PET and MRI data were acquired for each participant. Acquisition and PET image reconstruction methods were described previously for both the Cambridge^8^ and WCM^7^ data. Some modifications were necessary to combine the datasets (further details in eMethods 1 in Supplement 1), alongside data kinetic modeling with reference tissue region of interest (ROI) in the pons^8^ and estimation of voxelwise nondisplaceable binding potential relative to nondisplaceable distribution volume (BP_ND_). Patients recruited in Cambridge additionally underwent fluid-attenuated inversion recovery imaging, used to manually delineate traumatic contusions using Analyze, version 14.0 (AnalyzeDirect), with reference to the other magnetic resonance sequences obtained (T_1_ weighted, T_2_ weighted, gradient echo).

T_1_-weighted images underwent statistical parametric mapping unified segmentation with SPM, version 12 (University College London), with light regularization (0.001), and forward-deformation fields were applied to bias-corrected and segmented images for spatial normalization into Montreal Neurological Institute standard space (MNI152). These fields were also applied to BP_ND_ maps, subsequently smoothed with an 8-mm full-width at half-maximum Gaussian kernel. For ROI analysis of FMZ BP_ND_, thalamic and frontal ROI normalized volume was extracted for inclusion as a covariate in the linear model to distinguish neuronal loss from gross volume loss. Further details are provided in eMethods 1 in Supplement 1.

### Statistical Analysis

Potential group differences in age (2-sample *t* test) and sex (χ^2^) were assessed between patients and control participants. Patients were additionally compared between recruitment sites for differences in injury severity and time from injury to scan (2-sample *t* tests). All imaging-derived variables described in this section were initially compared between control and patient groups at a Benjamini-Hochberg false discovery rate (FDR)–corrected *P* < .05 with covariates of age, sex, and acquisition site, unless stated otherwise.

Voxelwise differences were first explored in SPM12 using a 2-sample *t* test at a significance threshold of *P* < .001 (uncorrected) at the voxel level and of *P* < .05 (familywise error corrected) at the cluster level to define minimum cluster size. Cerebrospinal fluid was excluded using the SPM12 tissue probability map (thresholded >0.75). Based on the voxelwise results, the left and right thalami and 7 thalamic nuclei per hemisphere were further investigated using an atlas of human thalamic nuclei,^9^ excluding 3 patients with visible thalamic lesions on MRI. Additionally, the frontal medial and paracingulate ROIs defined by the Harvard-Oxford probabilistic atlas were investigated, excluding 11 patients with visible frontal contusions on MRI. The mean FMZ BP_ND_ was extracted within each ROI and compared between patient and control groups using 2-sample *t* tests, additionally including a normalized mask volume covariate to differentiate neuronal loss vs gross volume loss. These extracted normalized thalamic volumes were additionally compared between patient and control groups.

Each variable found to have a statistically significant difference between control and patient groups was then related to available outcomes. These outcomes included Glasgow Outcome Scale (GOS) scores, 36-Item Short Form Health Survey (SF-36) scores on 7 domains, animal fluency (ie, the number of unique animals named) at 60 and 90 seconds and sustained fluency in the last 30 seconds (90 − 60), and cognitive assessments from the Cambridge Neuropsychological Test Automated Battery (CANTAB). Specific test measures were particularly chosen if found informative of TBI outcome in a recent large-scale outcome study.^10^ Analyses were only conducted in the Cambridge TBI cohort without thalamic lesions (n = 15), as similar outcomes were not available for patients with TBI in the WCM cohort. Covariates of age, sex, initial injury severity (Glasgow Coma Scale score), time from injury to scan, and normalized ROI volume were included in comparisons of mean ROI FMZ BP_ND_. All tests were FDR corrected for multiple comparisons at *P* < .05 within test.

Finally, we hypothesized that thalamic regions with reduced FMZ BP_ND_ in TBI mirrored the cortical contusions exhibited by our specific cohort. Thalamic nuclei have known connectivity to differential regions of the cortex^11^; thus, cortical contusion ROIs would be structurally connected to different regions of the thalamus prior to injury, as they are in healthy control participants. Analyses were conducted with data for patients in the TBI cohort collected in Cambridge for which contusion mapping was performed, who also did not present any thalamic lesions (n = 15).

A healthy average template constructed from 1065 healthy adult scans^12^ was used as a proxy for preinjury normative structural connectivity. Deterministic fiber tracking (detailed in eMethods 2 in Supplement 1) estimated total numbers of thalamic nucleus-to-contusion tracts, which were normalized by the respective total number of nucleus-to-cortex tracts, to produce a probability of nucleus-to-contusion structural connectivity for each patient. An example is given in eFigure 5 in Supplement 1 for visualization. Patients for whom tractography algorithms found 0 nucleus-to-contusion tracts were excluded for that analysis (n = 2 excluded; final cohort, n = 13). Thalamic nuclei FMZ BP_ND_ values were adjusted for sex, age, and normalized ROI volume, and then correlated with nucleus-to-contusion connectivity probability using Pearson correlation (FDR corrected) at *P* < .05. Statistical analyses were conducted using RStudio, version 4.1.2 (Posit, PBC).

## Results

A total of 24 patients with chronic TBI and 33 healthy control participants underwent FMZ PET. Patients with TBI had a median time of 29 (range, 7-95) months from injury to scan, their mean (SD) age was 39.2 (12.3) years, and there were 18 men (75.0%) and 6 women (25.0%). Control participants had a mean (SD) age of 47.6 (20.5) years, and there were 23 men (69.7%) and 10 women (30.3%). The patient and control groups did not differ in terms of age or sex, nor did these variables differ between patients with TBI recruited in Cambridge and WCM (Table 1). However, substantially more patients with TBI were recruited in Cambridge vs WCM (n = 19 vs 5), with more severe initial injury and greater time from injury to imaging. All patients demonstrated evidence of traumatic injury on acute neuroimaging. Demographic information and statistical results for all groups are presented in Table 1.

**Table 1.** Summary of Demographic and Clinical Characteristics

Characteristic	Overall, No. (%)		Statistic	*P* value	Patients with TBI, No. (%)		Statistic	*P* value
	Control (n = 33)	Patients with TBI (n = 24)			Site			
					WCM (n = 5)	Cambridge (n = 19)		
Age, mean (SD) [range], y	45.5 (14.5) [22-71]	39.2 (12.3) [19-66]	*t*_55_ = 1.8	.08	47.6 (20.5) [34-58]	36.9 (11.9) [19-66]	*t*_22_ = 1.9	.10
Sex								
Male	23 (69.7)	18 (75.0)	χ_1_<0.01	>.99	3 (60.0)	15 (78.9)	χ_1_<0.01	.96
Female	10 (30.3)	6 (25.0)			2 (40.0)	4 (21.1)		
Site								
Cambridge	16 (48.5)	19 (79.2)	χ_1_ = 4.3	.04	NA	NA	NA	NA
WCM	17 (52.5)	5 (20.8)			NA	NA	NA	NA
Glasgow Coma Scale score, median (range)^a^	NA	NA	NA	NA	14 (9-15)	6 (3-12)	NA	.02^b^
Injury mechanism								
Road traffic collision	NA	14 (58.3)	NA	NA	3 (60.0)	11 (57.9)	NA	>.99^b^
Fall	NA	7 (29.1)	NA	NA	2 (40.0)	5 (26.3)	NA	
Assault	NA	1 (4.2)	NA	NA	0	1 (5.3)	NA	
Other	NA	1 (4.2)	NA	NA	0	1 (5.3)	NA	
Unknown	NA	1 (4.2)	NA	NA	0	1 (5.3)	NA	
Time from injury to scan, median (range),^c^ mo	NA	NA	NA	NA	14 (13-20)	36 (7-95)	*t*_22_ = 4.1	<.001
Glasgow Outcome Scale–Extended,^d^ median (range)	NA	NA	NA	NA	7 (7-8)	6 (3-8)	NA	.003^b^
Glasgow Outcome Scale,^e^ median (range)	NA	NA	NA	NA	5 (5-5)	4 (3-5)^f^	NA	.12^b^

Abbreviations: NA, not applicable; TBI, traumatic brain injury; WCM, Weill Cornell Medicine.

^a^ Overall for patients with TBI, median score, 8 (range, 3-15).

^b^ Fisher exact *P* value.

^c^ Overall for patients with TBI, median time, 29 months (range, 7-95).

^d^ Overall for patients with TBI, median score, 6 (range, 3-8).

^e^ Overall for patients with TBI, median score, 4 (range, 3-5).

^f^ Missing 1.

### Local Reductions in FMZ BP_ND_

**Figure 1.**
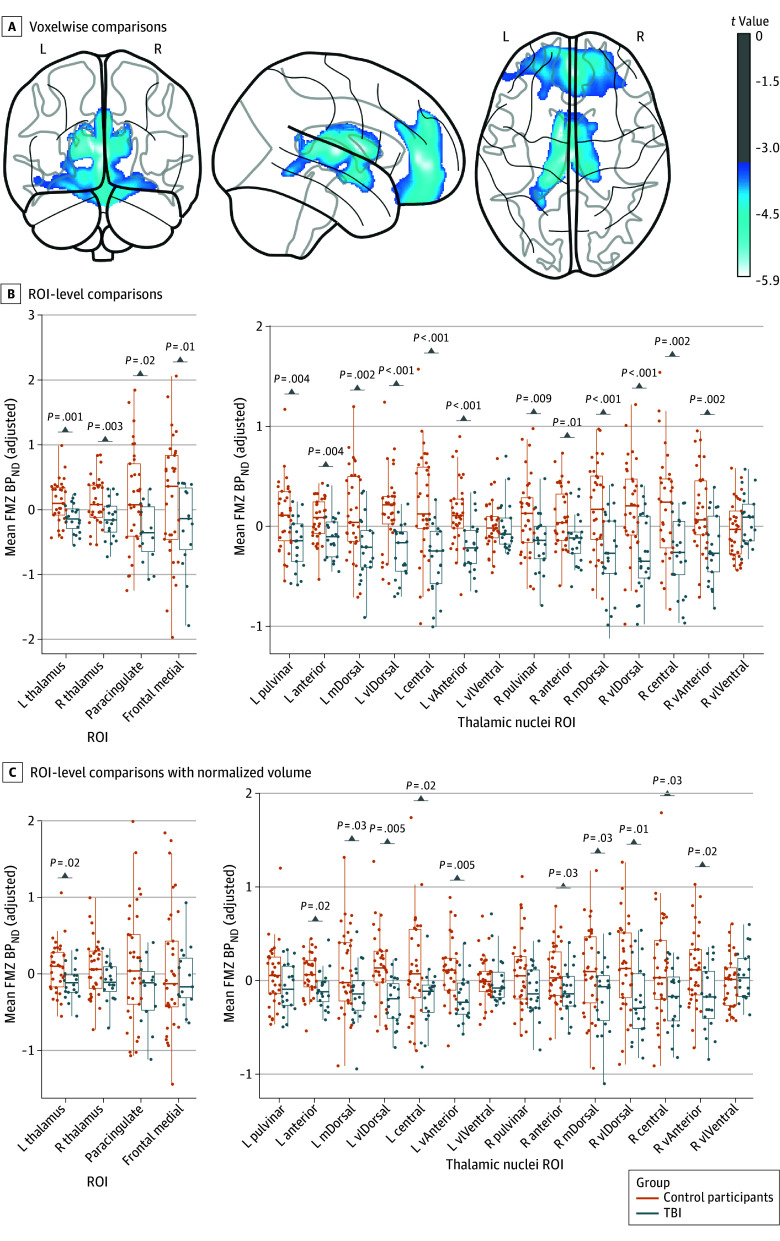
[^11^C]Flumazenil (FMZ) Nondisplaceable Binding Potential Relative to Nondisplaceable Distribution Volume (BP_ND_) Reduction in Chronic Traumatic Brain Injury (TBI) A, Voxelwise comparisons of FMZ BP_ND_ between the control and TBI groups, with all patients included (n = 24). The color bar shows *t* values surviving significance thresholds of *P* < .001 (uncorrected, voxel level) and *P* < .05 (familywise error, cluster level). B and C, Region of interest (ROI)-level comparisons between control participants and patients with TBI, with statistically significant differences surviving false-discovery rate correction indicated with *P* values. The y-axis of mean FMZ BP_ND_ is adjusted for covariates. Panel B presents comparisons when including covariates of age, sex, and research site. Panel C demonstrates the remaining statistically significant differences when normalized ROI volume is additionally included as a covariate in the linear model. Patients with TBI were excluded from each comparison in panels B and C if they presented a contusion within that region (n = 3 excluded for thalamic ROIs and n = 11 excluded in frontal ROIs). L indicates left; m, medio; R, right; vl, ventral-lateral.

In voxelwise comparisons, we found no regions with increased FMZ BP_ND_ in the patient group but 2 main clusters of decreased FMZ BP_ND_ were identified: (1) the thalami and (2) the frontal medial and paracingulate cortices. These results are presented as voxelwise t-values in Figure 1A. These regions were consistently identified when excluding subgroups of patients with injured brain regions (frontal contusions, n = 11; and thalamic contusions, n = 3) or when excluding the WCM cohort with milder TBI (n = 5) (eFigure 1 in Supplement 1).

Regions of interest in the thalamus and its nuclei, as well as in the frontal medial and paracingulate cortices, were assessed for group differences in FMZ BP_ND_, excluding patients with ROI lesions. Statistically significant differences between patients and control participants are shown in Figure 1B. Only specific thalamic ROIs remained statistically significant when normalized ROI volume was included in the model (Figure 1C and Table 2). Plots of unadjusted mean FMZ BP_ND_ are presented in eFigure 2 in Supplement 1.

**Table 2.** Group Comparisons of Regional FMZ BP_ND_ Between Control and Patient Cohorts

ROI	FMZ BP_ND_ for control group vs TBI group^a^	*P* value^b^	Mean difference (95% CI)
Left thalamus	8.69	.02	−0.19 (−0.34 to −0.04)
Pulvinar	3.07	.10	−0.13 (−0.29 to 0.03)
Anterior	7.35	.02	−0.16 (−0.28 to −0.04)
Mediodorsal	6.34	.03	−0.23 (−0.44 to −0.02)
Ventral-lateral dorsal	14.90	.005	−0.36 (−0.53 to −0.18)
Central	8.22	.02	−0.32 (−0.55 to −0.08)
Ventral anterior	13.61	.005	−0.30 (−0.46 to −0.14)
Ventral-lateral ventral	0.06	.81	−0.02 (−0.16 to 0.13)
Right thalamus	4.56	.05	−0.17 (−0.34 to 0.01)
Pulvinar	3.98	.06	−0.18 (−0.36 to 0.00)
Anterior	5.44	.03	−0.18 (−0.34 to −0.03)
Mediodorsal	6.58	.03	−0.26 (−0.50 to −0.03)
Ventral-lateral dorsal	10.09	.01	−0.36 (−0.61 to −0.12)
Central	6.11	.03	−0.29 (−0.53 to −0.04)
Ventral anterior	8.66	.02	−0.30 (−0.51 to −0.09)
Ventral-lateral ventral	1.09	.32	0.07 (−0.08 to 0.23)
Paracingulate cortex	1.34	.25	−0.32 (−0.68 to 0.03)
Frontal medial cortex	5.09	.05	−0.10 (−0.45 to 0.26)

Abbreviations: BP_ND_, nondisplaceable binding potential relative to nondisplaceable distribution volume; FMZ, [^11^C]flumazenil; ROI, region of interest; TBI, traumatic brain injury.

^a^ Includes covariates of age, sex, research site, and normalized ROI volume. *F* values are *F*_1,47_ for left thalamus ROIs and right thalamus ROIs and *F*_1,39_ for paracingulate and frontal medial cortices.

^b^ All false-discovery rate corrected.

### Association of Chronic Thalamic Selective Neuronal Loss With Outcome

**Figure 2.**
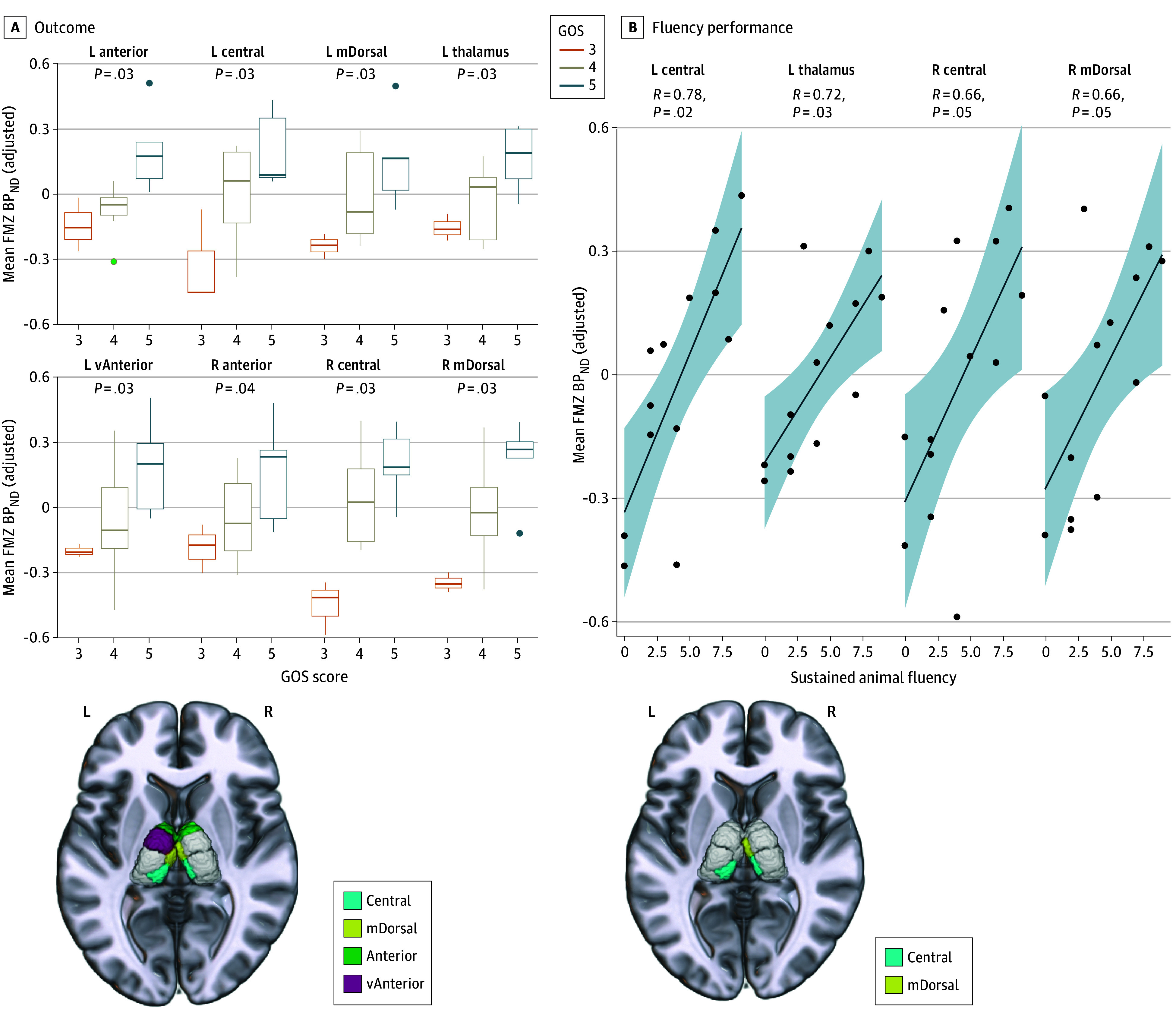
Association Between [^11^C]Flumazenil (FMZ) Nondisplaceable Binding Potential Relative to Nondisplaceable Distribution Volume (BP_ND_) and Chronic Outcome A and B, Statistically significant correlations between mean FMZ BP_ND_ within a region of interest (ROI) and outcome for Glasgow Outcome Scale (GOS) score (A) and animal fluency in 60 to 90 seconds (B). Full test results are presented in the Results. The y-axis of mean FMZ BP_ND_ is adjusted for covariates of age, sex, research site, and normalized ROI volume. Each test included patients with nonthalamic contusions (n = 15) with that outcome available (n = 2 patients did not complete animal fluency due to fatigue). Corresponding thalamic view highlights associated nuclei in panels A and B, respectively. L indicates left; m, medio; R, right; v, ventral.

Regions of interest with group differences (after inclusion of normalized volume) were associated with outcome (n = 15 patients available). Associations were found between lower GOS score and decreased FMZ BP_ND_ in the left thalamus (τb = 0.50, *P* = .03) and in the bilateral central (left: τb = 0.52, *P* = .03; and right: τb = 0.57, *P* = .03), bilateral mediodorsal (left: τb = 0.52, *P* = .03; and right: τb = 0.59, *P* = .03), bilateral anterior (left: τb = 0.61, *P* = .03; and right: τb = 0.48, *P* = .04), and left ventral anterior (τb = 0.5, *P* = .03) thalamic nuclei. Three patients had a GOS score of 3, 7 had a score of 4, and 5 had a score of 5. We next found an association between lower FMZ BP_ND_ in the right central thalamus and subscales of the SF-36: we observed lower scores for mental health (*r*_13_ = 0.71, *P* = .03) and for the subgroup reporting role limitations due to emotional problems (mean difference, −0.44 [95% CI, −0.74 to −0.13]; *F*_13_ = 11.8, *P* = .048). Additionally, we found an association between decreased performance on the animal fluency task (n = 2 of 15 patients did not complete it due to fatigue) and decreased FMZ BP_ND_ in the left thalamus (*r*_11_ = 0.72, *P* = .03) and in the bilateral central (left: *r*_11_ = 0.78, *P* = .02; and right: *r*_11_ = 0.66, *P* = .047) and right mediodorsal (*r*_11_ = 0.66, *P* = .047) thalamic nuclei. Interestingly, this association was only found with task performance in the last 30 seconds (ie, 60-90 seconds), but not if considering the total number of animals named in either sum time period (ie, 60 or 90 seconds). We did not find any associations with CANTAB measures after correcting for multiple comparisons. These findings are summarized in Figure 2, and results from all comparisons are provided in eFigure 3 in Supplement 1. Interestingly, despite finding statistically significant gross volume reductions in 10 of 16 thalamic nuclei between the TBI and control groups, these changes were not associated with outcome, shown in eFigure 4 in Supplement 1.

### Chronic Thalamic Damage and Cortical Damage

**Figure 3.**
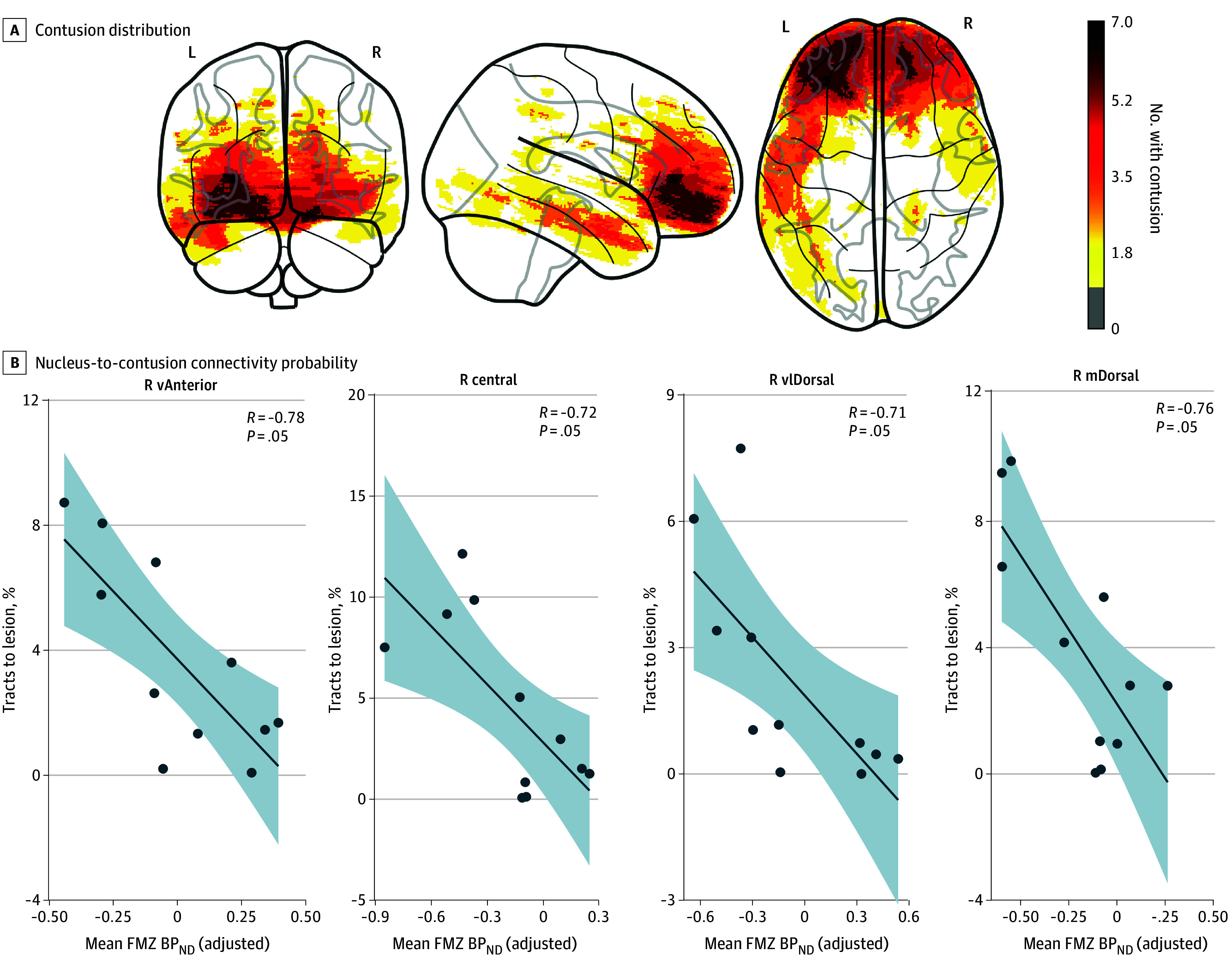
Association Between [^11^C]Flumazenil (FMZ) Nondisplaceable Binding Potential Relative to Nondisplaceable Distribution Volume (BP_ND_) and Contusion Structural Connectivity A, Traumatic contusion masks summed across the group with traumatic brain injury. Values indicate the number of patients with a contusion in that region (ie, a value of 4 indicates 4 patients exhibit contusions at this location). B, Statistically significant negative correlations between FMZ BP_ND_ and structural connectivity probability, where each point is an individual. Individuals were included if they did not present a thalamic lesion with contusion mask (n = 15) and were successful at producing some tracts between the respective thalamic nucleus and their contusion mask. Results indicate a mirroring effect between cortical damage and chronic thalamic neuronal loss. The x-axis of mean FMZ BP_ND_ is adjusted for covariates. Prefix m indicates medio; v, ventral.

Diffusion tensor imaging further related regions of cortical damage to regions of thalamic selective neuronal loss. A summary of patient contusion distribution is presented in Figure 3A. Correlations between FMZ BP_ND_ (adjusted for all covariates) and nucleus-to-contusion structural connectivity probability (eFigure 5 in Supplement 1) revealed negative associations, such that reduced FMZ BP_ND_ in all thalamic nuclei was correlated with greater likelihood of preinjury structural connectivity of that thalamic nucleus to each contusion ROI. These correlations survived FDR correction in 4 nuclei: right mediodorsal (*R*9 = −0.76, *P* = .048), right central (*R*9 = −0.72, *P* = .048), right ventral anterior (*R*9 = −0.78, *P* = .048), and right ventral-lateral dorsal (*R*9 = −0.71, *P* = .048), shown in Figure 3B. Results for all nuclei are shown in eFigure 6 in Supplement 1. Analyses were replicated with a local independent cohort (eMethods 2 in Supplement 1, with results presented in eFigure 7 in Supplement 1) for additional validation.

## Discussion

To our knowledge, this is the largest cross-sectional study of patients with TBI to use FMZ PET to examine whether chronic TBI is characterized by selective thalamic neuronal loss not solely attributable to gross volume loss and in absence of evidence for traumatic injury. This loss was present in all TBI severities extending up to 95 months (7.9 years) postinjury, highlighting the enduring consequences of TBI in thalamic integrity. Our findings suggest that such loss was further associated with worse functional, cognitive, and emotional outcomes. We additionally showed mirroring of thalamic selective neuronal loss and cortical contusion location, which may be associated with injury mechanisms of transneuronal degeneration. Thus, we propose that selective thalamic vulnerability may have lifelong neuronal consequences with relevance to long-term outcome, particularly focused on the central and medial thalami.

The thalamus is highly vulnerable to forces experienced during primary injury,^13^ and it shows a unique perpetuation of inflammatory markers postinjury suggestive of ongoing injury processes.^14,15^ Previous studies using MRI have found subacute thalamic impairment to be associated with poor long-term outcome across all severities of TBI,^16,17^ and thalamic volume loss up to 6 months postinjury has been suggested to link the injury event to ongoing disease, above all other brain regions.^17^ In our study, thalamic injury extended well beyond these time points (over and above gross volume loss) and was suggestive of late functional outcome, making it integral to our understanding of long-term disease after TBI. Accordingly, these findings suggest that FMZ PET has potential to improve understanding of the ongoing neuronal burden after TBI, due to greater sensitivity and specificity to neuronal loss than routine imaging.

Crucially, we observed that medial/central thalamic nuclei were consistently associated with injury and outcome. These nuclei have consistently shown evidence of injury in histologic studies of TBI,^18^ gross volume loss in chronic TBI,^19^ and volume change over the first 6 months after injury associated with scores on the Glasgow Outcome Scale–Extended and recovery of consciousness.^20^ This central aspect of the thalamus is also thought to be important in the maintenance and recovery of consciousness in anesthesia and disorders of consciousness,^21,22^ and it displays the shortest point-to-point connections in the mammalian corticothalamic system,^23^ providing a natural model for their sustaining proportionately greater deafferentation in multifocal injury. It therefore stands that these central and medial thalamic regions have clear vulnerability to injury, they are an integral part of healthy brain function and consciousness, and evidence of injury in such regions is important for recovery after brain injury.

We further propose that chronic vulnerability of these nuclei may be associated with transneuronal degeneration, due to mirroring of thalamic neuronal loss and cortical damage. Numerous animal models have demonstrated thalamic damage at later time points than cortical damage,^14,15^ consistent with retrograde neuronal injury and apoptosis, even mirroring the location of cortical damage after TBI to its structurally connected thalamic regions.^24^ In humans, patients with TBI with nonthalamic contusions have also shown reduced thalamic volumes than those without, independent of injury severity and ventricular volume.^25^ In this study, we provided specificity to this hypothesis in humans. Although we were unable to discern directionality of this mirroring effect in our cohort, previous literature^14,15,24^ suggests that secondary transneuronal thalamic degeneration may underlie selective vulnerability of medial thalamic nuclei in our cohort.

Moreover, such medial thalamic selective neuronal loss was indicative of worse long-term outcomes in this study, which were not identified using thalamic volume measures, including sustained fluency. Categorical fluency (related to domains of language and executive function) shows poor performance in Alzheimer disease^26^ and greater attrition over task duration in patients with mild cognitive impairment.^27^ Similarly, patients with chronic TBI have shown performance decline with prolonged attention and task demands in classical neuropsychological tasks, partially mediated in some patients with methylphenidate treatment.^28,29^ Thus, there is potential clinical utility of time-related performance decline on the animal fluency task for TBI and mild cognitive impairment, and its association with later risk of Alzheimer disease, which can be linked to thalamic neuronal loss. This finding suggests that targeting existing and novel interventions based on the thalamus may have long-term therapeutic benefit, which showed promising results in a recent rat model.^30^ To translate such findings into improved outcome and long-term benefit for patients will require improved understanding of the therapeutic window for both primary and secondary thalamic injury.

### Limitations

This study has several limitations. The data were based on 2 independent cohorts that were retrospectively collated with nonconsecutive recruitment (owing to convenience and logistics), which makes generalizability difficult to assess. There were also differences in some imaging acquisition parameters, the severity of initial injury, and the outcome measures obtained, thereby limiting our sample sizes for outcome group comparisons. The PET data were harmonized across the 2 sites as far as possible; however, some differences remained, such as the attenuation correction method. Therefore, to minimize any effect of such differences, acquisition site was consistently included as a covariate in all analyses. Second, consistent with the typical pattern of injuries found after TBI, our data demonstrated a preponderance of frontal and temporal contusions (Figure 3A). Therefore, our sample sizes when investigating frontal and temporal brain regions were limited after exclusion of patients with contusions in such regions, which may have restricted our ability to replicate significantly reduced frontal FMZ BP_ND_.^7^ This pattern of contusions may have similarly limited our ability to find mirroring of thalamic loss in all nuclei, particularly left-hemisphere nuclei, which could be due to inherent network asymmetries often associated with neurodegenerative diseases^31^ or attributable to our cohort not presenting contusion in structurally connected cortical regions. Third, although our study more than doubles previous sample sizes studying FMZ PET in chronic TBI,^4,5,7^ the numbers remain small, and our results should be replicated in a larger sample in future studies. Finally, we can merely speculate on the mechanism responsible for late secondary thalamic neuronal loss; this would require a combined longitudinal MRI and PET study of thalamic FMZ BP_ND_ progression against cortical contusion location to fully elucidate.

## Conclusions

On the basis of the findings of this cross-sectional study, we propose that selective thalamic vulnerability may have chronic neuronal consequences with relevance to long-term outcome, emphasizing the ongoing disease that results from acute TBI. [^11^C]Flumazenil PET showed greater sensitivity to outcome measures than thalamic volume determined from MRI and may advance our understanding of the full neuronal burden of injury across brain regions that initially appear healthy. Primary and evolving secondary consequences of thalamic injury should be further investigated to promote more informed prognostic and therapeutic modeling and targeted patient care.

## Notes

Supplement 1. **eMethods 1.** Image Acquisition and Processing **eFigure 1.** Voxelwise Comparisons of [^11^C]Flumazenil (FMZ) Nondisplaceable Binding Potential Relative to Nondisplaceable Distribution Volume (BP_ND_) in Patient Subgroups **eFigure 2.** Plots of Unadjusted Mean FMZ BP_ND_ **eFigure 3.** Summary of Tests Between Thalamic Region of Interest (ROI) FMZ and Outcome Measures **eFigure 4.** Comparisons of Thalamic Volume **eFigure 5.** Visualization of Percentage Tracts to Contusion Calculation **eFigure 6.** Correlation Between Thalamic Nucleus FMZ BP_ND_ and Nucleus-to-Contusion Structural Connectivity Probability for All Thalamic Nuclei **eMethods 2.** Relationship Between Thalamic Nucleus FMZ BP_ND_ and Nucleus-to-Contusion Structural Connectivity **eFigure 7.** Correlation Between Thalamic Nucleus FMZ BP_ND_ and Nucleus-to-Contusion Structural Connectivity Probability Determined Using a Locally Acquired Diffusion Tensor Imaging Dataset for All Thalamic Nuclei
eReferences

Supplement 2.
Data Sharing Statement
