# Supplementary material for: Outcomes and Mechanisms Associated With Selective Thalamic Neuronal Loss in Chronic Traumatic Brain Injury
Source: JAMA Netw Open. 2024 Aug 6;7(8):e2426141. doi: 10.1001/jamanetworkopen.2024.26141 (PMC11304117; doi:10.1001/jamanetworkopen.2024.26141)
Supplement: Supplement 1. — eMethods 1. Image Acquisition and Processing eFigure 1. Voxelwise Comparisons of [11C]Flumazenil (FMZ) Nondisplaceable Binding Potential Relative to Nondisplaceable Distribution Volume (BPND) in Patient Subgroups eFigure 2. Plots of Unadjusted Mean FMZ BPND eFigure 3. Summary of Tests Between Thalamic Region of Interest (ROI) FMZ and Outcome Measures eFigure 4. Comparisons of Thalamic Volume eFigure 5. Visualization of Percentage Tracts to Contusion Calculation eFigure 6. Correlation Between Thalamic Nucleus FMZ BPND and Nucleus-to-Contusion Structural Connectivity Probability for All Thalamic Nuclei eMethods 2. Relationship Between Thalamic Nucleus FMZ BPND and Nucleus-to-Contusion Structural Connectivity eFigure 7. Correlation Between Thalamic Nucleus FMZ BPND and Nucleus-to-Contusion Structural Connectivity Probability Determined Using a Locally Acquired Diffusion Tensor Imaging Dataset for All Thalamic Nuclei eReferences [file jamanetwopen-e2426141-s001.pdf]

Supplementary Online Content

Woodrow RE, Grossac J, Hong YT, et al. Outcomes and mechanisms associated with selective thalamic neuronal loss in chronic traumatic brain injury. *JAMA Netw Open*. 2024;7(8):e2426141. doi:10.1001/jamanetworkopen.2024.26141

eMethods 1. Image Acquisition and Processing

eFigure 1. Voxelwise Comparisons of [^{11}C]Flumazenil (FMZ) Nondisplaceable Binding Potential Relative to Nondisplaceable Distribution Volume (BP_{ND}) in Patient Subgroups

eFigure 2. Plots of Unadjusted Mean FMZ BP_{ND}

eFigure 3. Summary of Tests Between Thalamic Region of Interest (ROI) FMZ and Outcome Measures

eFigure 4. Comparisons of Thalamic Volume

eFigure 5. Visualization of Percentage Tracts to Contusion Calculation

eFigure 6. Correlation Between Thalamic Nucleus FMZ BP_{ND} and Nucleus-to-Contusion Structural Connectivity Probability for All Thalamic Nuclei

eMethods 2. Relationship Between Thalamic Nucleus FMZ BP_{ND} and Nucleus-to-Contusion Structural Connectivity

eFigure 7. Correlation Between Thalamic Nucleus FMZ BP_{ND} and Nucleus-to-Contusion Structural Connectivity Probability Determined Using a Locally Acquired Diffusion Tensor Imaging Dataset for All Thalamic Nuclei

eReferences

This supplementary material has been provided by the authors to give readers additional information about their work.

© 2024 Woodrow RE et al. *JAMA Network Open.*

eMethods 1. Image Acquisition and Processing

Cambridge

The MRI protocol included high-resolution 3D volume T1-weighted (T1w), T2-weighted and fluid-attenuation inversion-recovery (FLAIR) sequences, acquired on a 3T whole body magnet (Medspec s300; Bruker, Ettlingen, Germany). T1w images were resized to voxels of 1 × 1 × 1 mm^3 and re-orientated to the AC-PC line. FMZ PET data were acquired in 3D mode on a GE Advance PET Scanner (GE Medical Systems, Waukesha, USA). Prior to FMZ injection a 15 min transmission scan using rotating Ge-68 rod sources was acquired to correct for photon attenuation. FMZ was produced using a methylation process[1], providing high specific activities (370–550 GBq/mmol). FMZ was injected intravenously as a bolus (418 ± 21 MBq) and data were acquired for 75 minutes post-injection (55 times frames: 18 × 5s, 6 × 15s, 10 × 30s, 7 × 60s, 4 × 150s and 10 × 300s). Images were reconstructed using the PROMIS 3D filtered back projection algorithm into 128 × 128 × 35 arrays with a voxel size of 2.34 × 2.34 × 4.25mm. Corrections were applied for randoms, dead time, normalisation, scatter, attenuation and sensitivity. Given that the duration of the WCM scans was 60 minutes, only images from the first 60 minutes of the Cambridge scans were used for subsequent analysis.

WCM

A 3T Siemens Prisma scanner with a 32-channel head coil was used to collect high-resolution 3D T1w images. Dynamic PET scans were performed on a Biograph mCT PET/CT scanner (Siemens Healthineers, Erlangen, Germany) over a period of 60 minutes from the injection of FMZ (407–595 MBq). For attenuation correction a low-dose CT scan was acquired. To match the Cambridge data, the list-mode PET data were histogrammed into the same time frames and were reconstructed

© 2024 Woodrow RE et al. *JAMA Network Open.*

using the same reconstruction algorithm –3D filtered back projection - into images with the same transaxial voxel size (2.34 × 2.34 mm). Corrections were applied for randoms, dead time, normalisation, scatter, attenuation and sensitivity. To harmonise the spatial resolution between the Cambridge (~ 6.5 mm FWHM) and WCM images (~ 5 mm FWHM), the WCM images were smoothed with a 4 mm FWHM isotropic Gaussian.

Image Processing

Kinetic modelling used a reference tissue ROI in the pons[2], drawn using Analyze 14.0 on 10 contiguous transverse planes of the re-orientated T1-weighted (T1w) image. PET images were realigned and co-registered to the corresponding T1w MR image using SPM12[3], and the reference tissue ROI was then projected to generate a time-activity curve. Voxel-wise binding potential relative to non-displaceable distribution volume (BP_{ND}) was determined with a basis function version of the simplified reference tissue model (RPM2[4]) with 100 basis functions for $0.001 \leq k_2 \leq 0.01$ sec^{-1}. A parametric map of k_2 was produced in order to determine a map of k_2', i.e. k_2 in the reference tissue. The median value of k_2' in voxels with $BP_{ND} \geq 0.5 \times BP_{ND}^{max}$ was used as a fixed parameter in the final estimation of voxel-wise BP_{ND}.

T1w images underwent SPM12 unified segmentation with light regularisation (0.001), and forward-deformation fields were applied to bias-corrected and segmented images for spatial normalisation into MNI152 standard space utilising 4th-degree B-spline interpolation. Each BP_{ND} map was spatially normalised using the forward-deformation field of the co-registered T1w image for global analysis (SPM12) and ROI analysis (ANTs). Spatially normalised BP_{ND} maps were smoothed with an 8 mm FWHM Gaussian kernel.

© 2024 Woodrow RE et al. *JAMA Network Open.*

ROI volume extraction

Each native T1w scan was corrected for scanner bias field inhomogeneities[5] and spatially normalised to the respective atlas template via affine and non-linear registration in ANTs[6]. To determine ROI volume in native space the inverse transformation was used to project ROIs from MNI template space to native T1w space with nearest neighbour interpolation. For each subject native T1w space ROI volumes were then normalised by total brain volume, estimated via automated brain extraction[7].

© 2024 Woodrow RE et al. *JAMA Network Open.*

eFigure 1. Voxelwise Comparisons of [^{11}C]Flumazenil (FMZ) Nondisplaceable Binding Potential Relative to Nondisplaceable Distribution Volume (BP_{ND}) in Patient Subgroups

Voxel-wise comparisons of FMZ BP_{ND} were repeated between controls and TBI sub-groups to assess the stability of the results. A) Moderate/severe TBI (n=19), B) TBI excluding those with frontal contusions (n=13), C) TBI excluding those with thalamic lesions (n=21). Colour bar indicates t-values surviving voxel-level and cluster-level thresholding for significance. These demonstrate the relative stability of frontal and thalamic regions of reduced FMZ BP_{ND} in chronic TBI, when compared to healthy controls. Smaller clusters of significant change in B may be attributed to smaller sample size and thus power to find an effect.

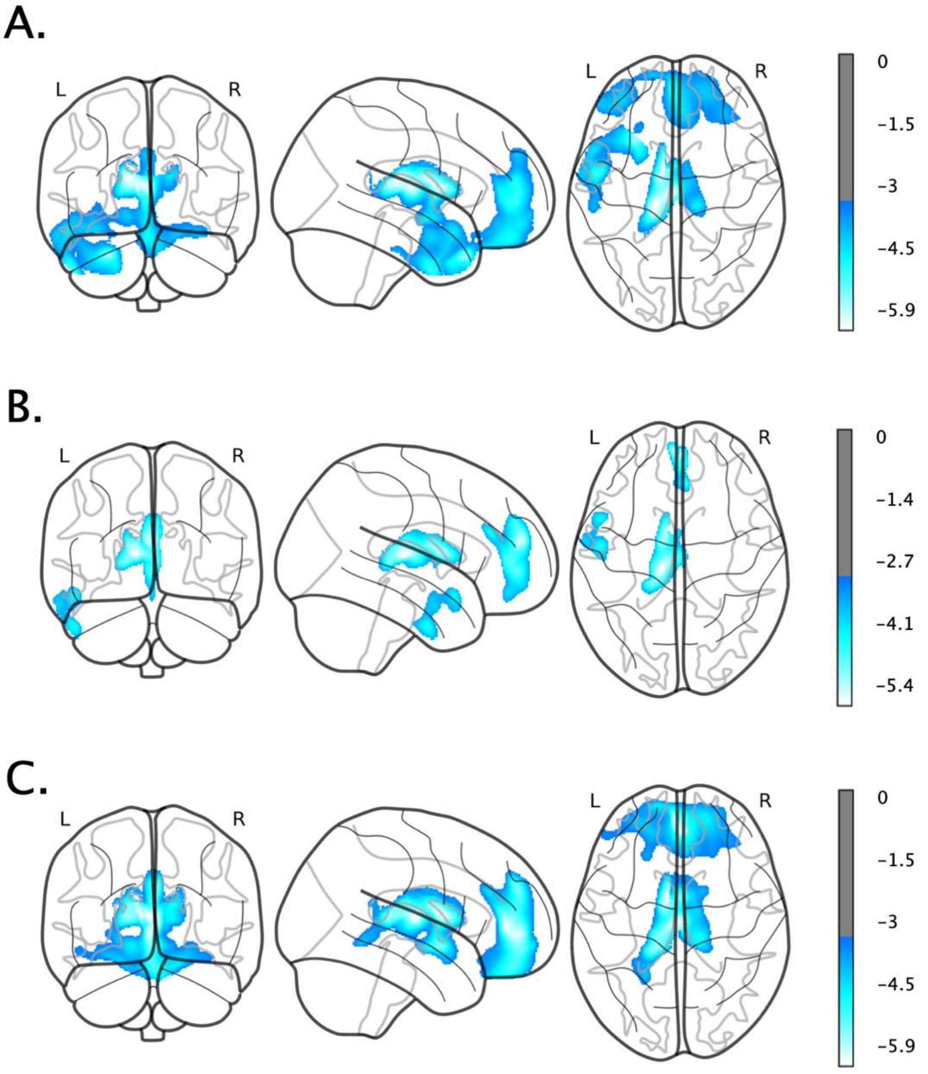

© 2024 Woodrow RE et al. *JAMA Network Open.*

eFigure 2. Plots of Unadjusted Mean FMZ BP_{ND}

Values are prior to inclusion of age, sex, research site, and ROI volume in the linear model. A shows comparison of healthy controls and patients. B shows comparisons of outcome in patients based on GOS.

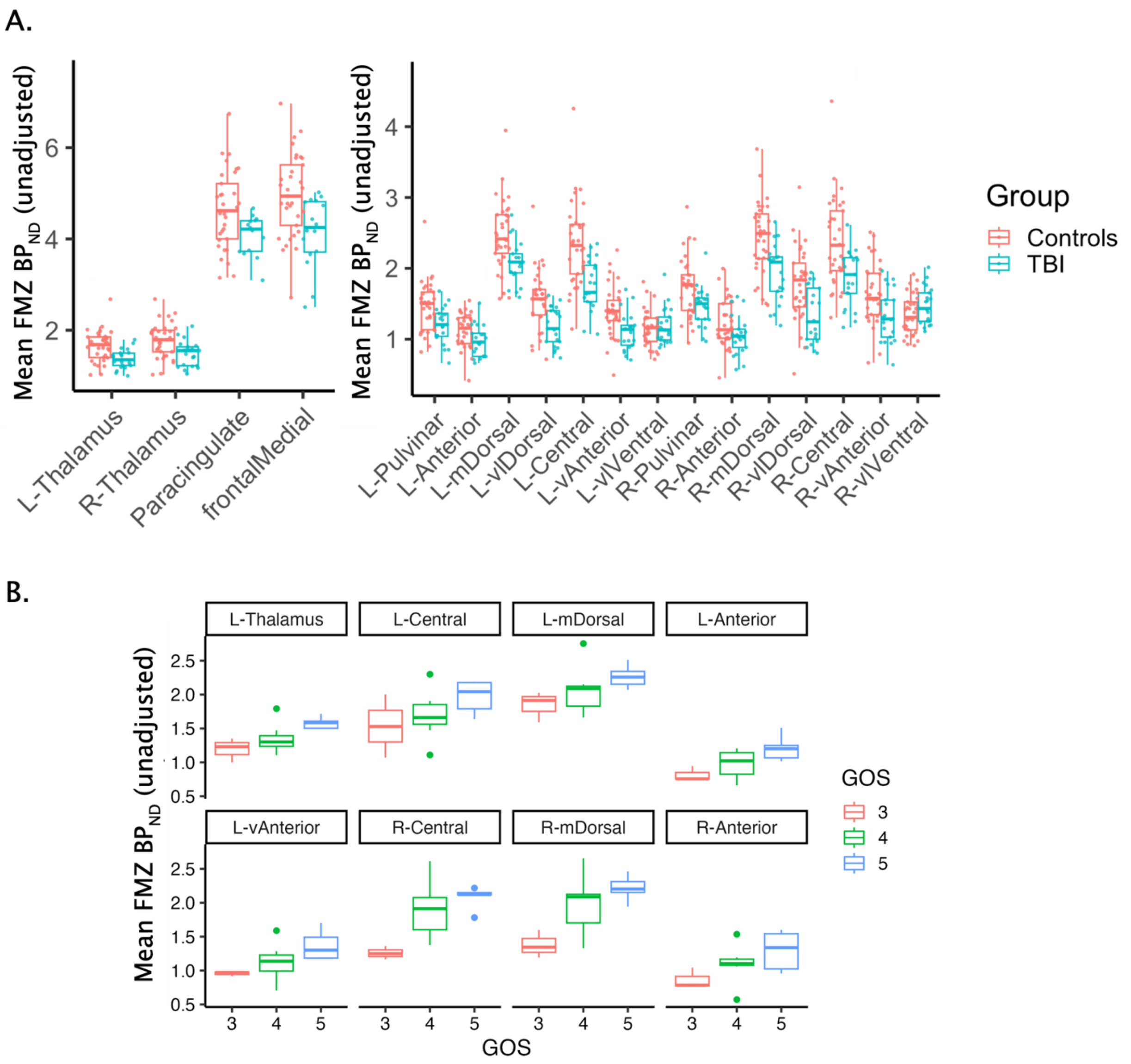

© 2024 Woodrow RE et al. *JAMA Network Open.*

eFigure 3. Summary of Tests Between Thalamic Region of Interest (ROI) FMZ BP_{ND} and Outcome Measures

Correlation matrix between thalamic ROI FMZ BP_{ND} and outcome measures. All data included covariates of age, sex, normalised ROI volume, baseline GCS, and days from injury to imaging, within the linear model. Colour bar and dot size indicates correlation statistic, with tests surviving FDR-correction ($p<0.05$) indicated with an asterisk. Outcome measures included are; Glasgow Outcome Scale (GOS), verbal fluency (FAS), animal fluency at 60, 90, and 60-90 seconds, SF-36 subscales (physical functioning (PF), role physical (RF), social functioning (SF), general health (GH), mental health (MH), bodily pain (BP), vitality (V), role emotional (RE)), CANTAB subscales (paired-associates learning (PAL), rapid visual processing (RVP), spatial working memory (SWM), intra-extra dimensional set shift (IED), pattern recognition memory (PRM), spatial recognition memory (SRM), spatial span (SSP)).

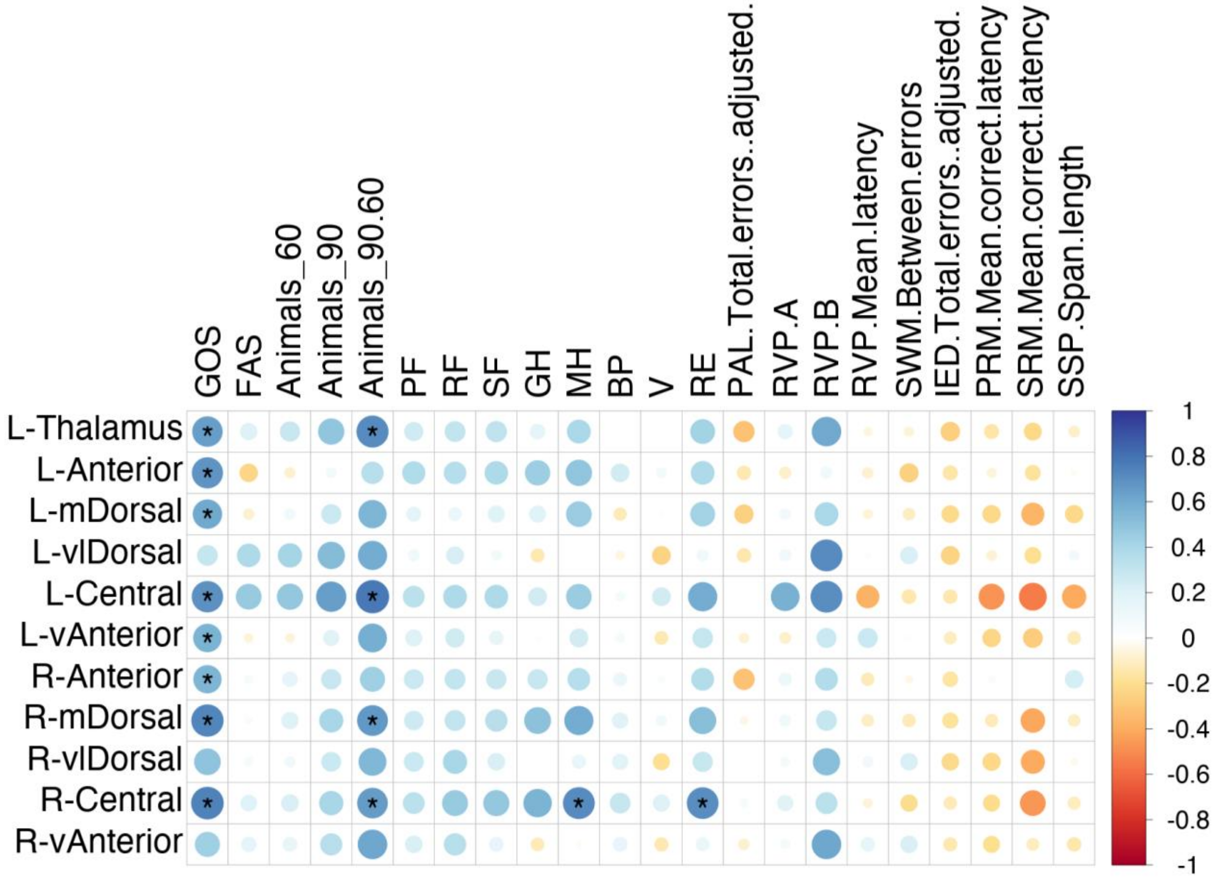

© 2024 Woodrow RE et al. *JAMA Network Open.*

eFigure 4. Comparisons of Thalamic Volume

A. Comparisons between control (HC) and TBI groups. Thalamic volumes are normalised by total brain volume, and comparisons include age, sex, and research site, in the linear model. Significant differences are shown at FDR-corrected p<0.05, where *= <0.05, **= <0.01, ***= <0.001.

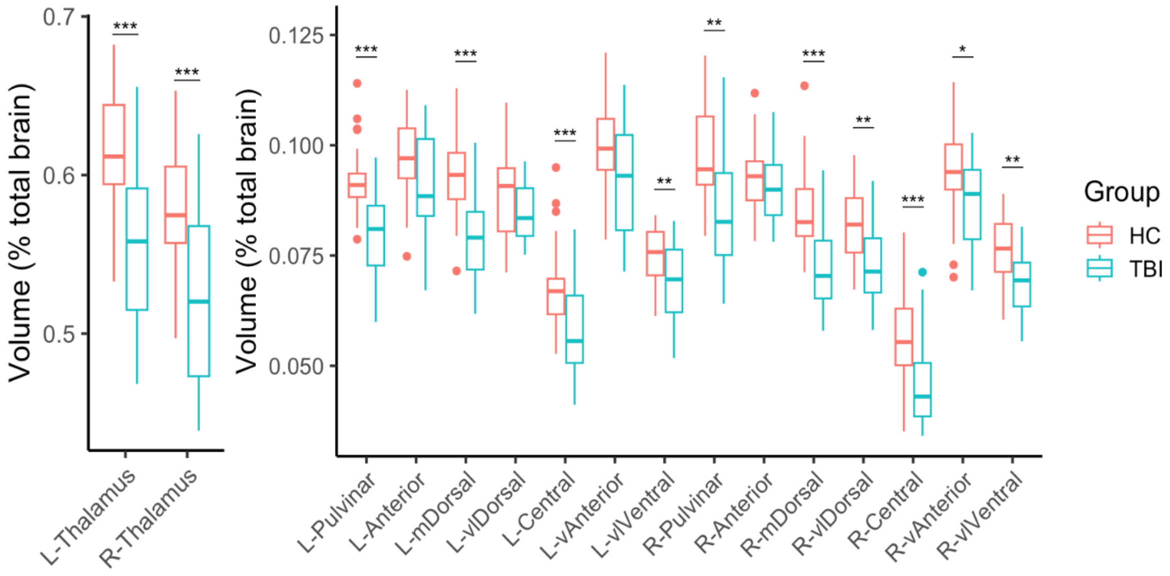

B. Relationships between thalamic volumes and outcome. All data included covariates of age, sex, baseline GCS, and days from injury to imaging, within the linear model. Colour bar and dot size indicate correlation statistic, with tests surviving FDR-correction (p<0.05) indicated with an asterisk.

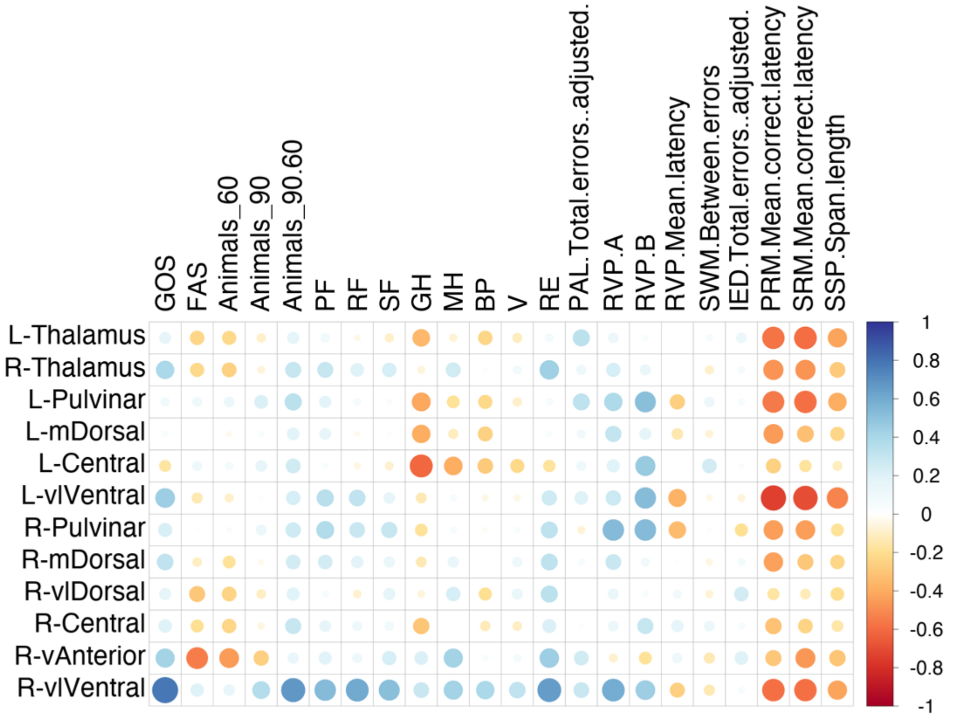

© 2024 Woodrow RE et al. *JAMA Network Open.*

eFigure 5. Visualization of Percentage Tracts to Contusion Calculation

Top: example tractography from thalamic nucleus to individual's contusion mask (blue), bottom: example tractography from thalamic nucleus to individual's cortical mask (blue). Both images are shown for the same individual, as example. Percentage of tracts from nucleus-to-contusion is (total tracts in B(top)) / (total tracts in B(bottom)) x 100.

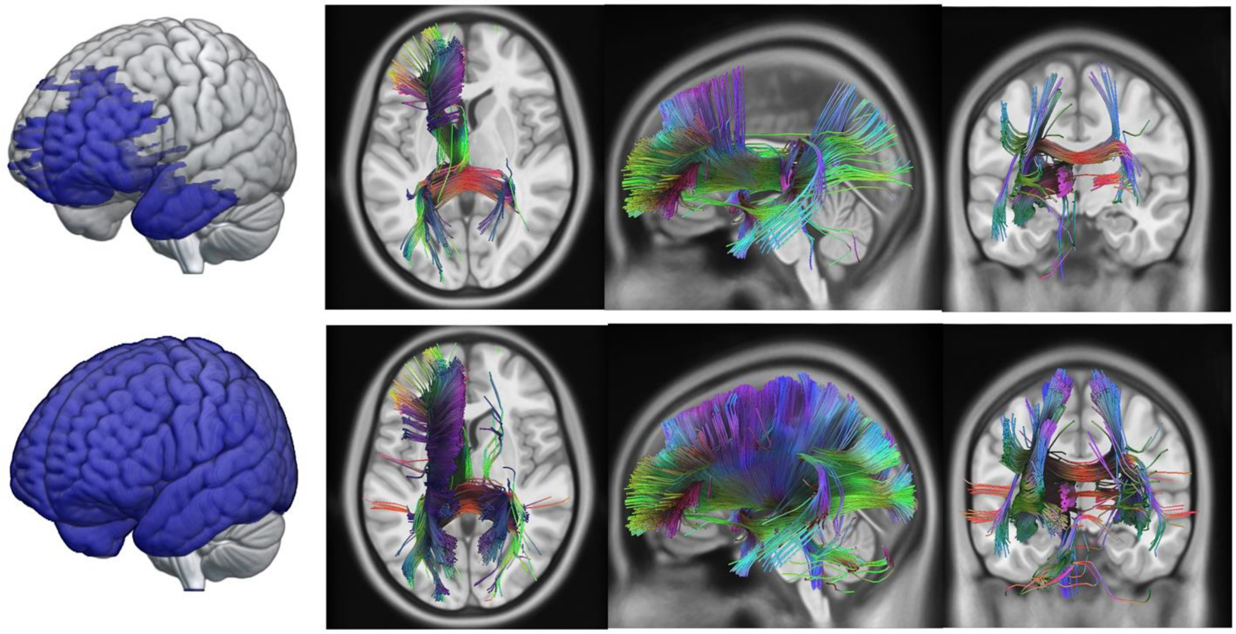

eFigure 6. Correlation Between Thalamic Nucleus FMZ BP_{ND} and Nucleus-to-Contusion Structural Connectivity Probability for All Thalamic Nuclei

Pearson's correlation between FMZ BP_{ND} and structural connectivity probability for all thalamic nuclei, where each point is an individual subject. All p-values are FDR-corrected. X-axes are adjusted for covariates.

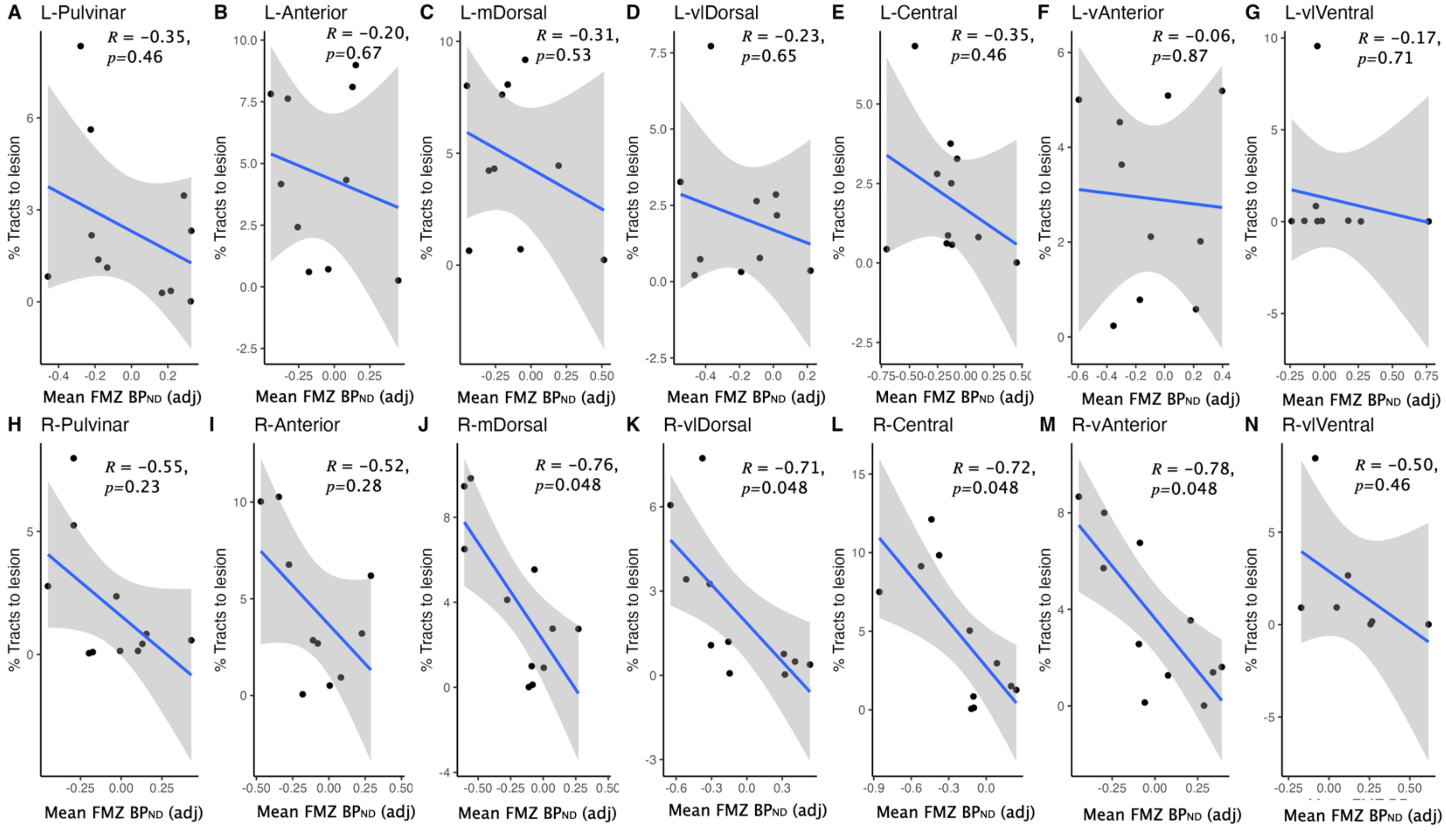

© 2024 Woodrow RE et al. *JAMA Network Open.*

eMethods 2. Relationship Between Thalamic Nucleus FMZ BP_{ND} and Nucleus-to-Contusion Structural Connectivity

Calculations of healthy control structural connectivity between thalamic nuclei and contusion masks were performed using a healthy average dataset (n=1065) in the main text. A deterministic fibre tracking algorithm(19) with augmented tracking strategies[8] was used to improve reproducibility. This involved 1,000,000 seeds in ROI-to-ROI tractography with standardised parameters[9]; tracts with length shorter than 30 or longer than 200 mm were discarded, anisotropy threshold was randomly selected, angular threshold was randomly selected from 15 degrees to 90 degrees, and step size was randomly selected from 0.5 voxel to 1.5 voxels. This was performed to estimate total number of thalamic nucleus-to-contusion tracts for each subject, which was normalised by the respective total number of nucleus-to-cortex tracts for that subject, to produce a probability of nucleus-to-contusion structural connectivity. This was calculated for each subject, for each thalamic ROI.

This methodology was further repeated with a locally collected dataset of n=18 healthy controls with diffusion tensor imaging (DTI), as described below.

Data acquisition & preprocessing

A Siemens Trio 3T MR system (Siemens Healthineers, Erlangen, Germany) was used to acquire the MRI data. For each subject, localiser images and 3D high resolution MPRAGE images (Relaxation Time (TR) 2300ms, Echo Time (TE) 2.98ms, Flip Angle 9°, field of view (FOV) $256mm^2 \times 256mm^2$) were acquired for use during pre-processing of diffusion MRI scans to assist spatial normalisation to Montreal Neurological Institute (MNI) space (https://www.mcgill.ca/neuro/). The diffusion MRI data (63 non-collinear directions, b=1000 s/mm^2 with one volume acquired without diffusion weighting (b=0), echo time 106ms, repetition time 1700ms, FOV $192mm^2 \times 92mm^2$, $2mm^3$ isotropic voxels) were acquired to investigate white matter tissue integrity.

© 2024 Woodrow RE et al. *JAMA Network Open.*

The diffusion-weighted imaging (DWI) scans were pre-processed using the MRtrix3 package (Tournier et al., 2019) (https://www.mrtrix.org/). Prior to the main sequence pre-processing, the data were denoised and residuals calculated. These indicate artifacts or distortions that may affect certain brain regions disproportionately. Data were unwarped and corrected for distortions, motion, and eddy currents. Following this, field inhomogeneities were corrected with the Advanced Normalisation Tools (ANTs) package (https://stnava.github.io/ANTs/). Brain masks were used to restrict analyses to only brain voxels.

DTI data were reconstructed from pre-processed DWIs using the MRtrix3 package. First, a basis function was constructed for each tissue type: grey matter (GM), white matter (WM), and cerebrospinal fluid (CSF). These basis functions were used to deconvolve and concatenate the fibre orientation distributions (FODs) for each tissue type. Finally, these were normalised for effects of intensity inhomogeneities.

Analysis

Each control subject was analysed in the same manner as in-text; a deterministic fibre tracking algorithm [10] was used with augmented tracking strategies [8] to improve reproducibility. This used 1,000,000 seeds in ROI-to-ROI tractography, whereby tracts with length shorter than 30 or longer than 200 mm were discarded. The anisotropy threshold was randomly selected, angular threshold randomly selected from 15 degrees to 90 degrees, and step size randomly selected from 0.5 voxel to 1.5 voxels. Using this method, we obtained the total number of nucleus-to-contusion tracts for each TBI subject with contusion mask available who also did not demonstrate evidence of a thalamic lesion (n=15), for each control (n=18). A mean value was then taken across the n=18 controls, and normalised by the respective total number of nucleus-to-whole brain cortex tracts, to produce a probability of nucleus-to-contusion structural connectivity. Subjects were excluded from analysis if no tracts were successfully produced between the contusion and thalamic nucleus. Probabilities calculated with the controls' data from local (n=18) and openly-available n=1065 datasets were compared with a Pearson's correlation, and were found to have high correspondence (R=0.91, p<0.001).

© 2024 Woodrow RE et al. *JAMA Network Open.*

Mean structural connectivity probabilities from the local control dataset were then correlated, as before, to the corresponding covariate-corrected thalamic nucleus FMZ BP_{ND}. Results are presented below in **eFig. 7**, prior to FDR-correction due to the smaller sample size of the local healthy control cohort (n=18) compared to the original healthy average dataset (n=1065). These results replicate those presented in-text, finding that all nuclei show a negative relationship whereby the same n=4 right-hemisphere nuclei show a significant mirroring effect between chronic thalamic neuronal loss and cortical damage, and additionally the right anterior nucleus. Thus, calculations from the two datasets show a high degree of correspondence and replication of results.

© 2024 Woodrow RE et al. *JAMA Network Open.*

eFigure 7. Correlation Between Thalamic Nucleus FMZ BP_{ND} and Nucleus-to-Contusion Structural Connectivity Probability Determined Using a Locally Acquired Diffusion Tensor Imaging Dataset for All Thalamic Nuclei

Pearson's correlation between FMZ BP_{ND} and structural connectivity probability for all thalamic nuclei, where each point is an individual subject. All p-values are FDR-corrected. X-axes are adjusted for covariates.

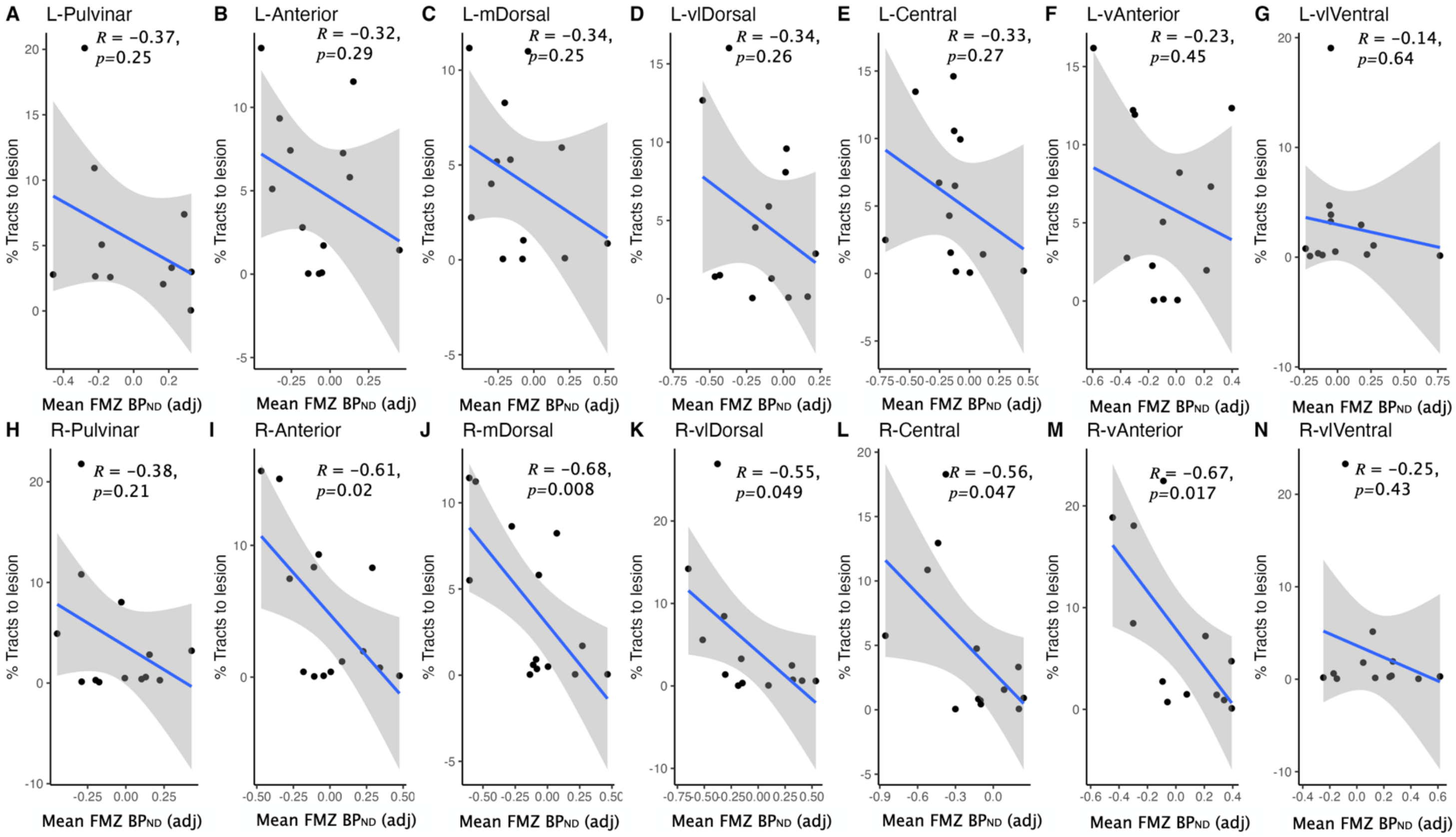

© 2024 Woodrow RE et al. *JAMA Network Open.*

eReferences

1. Cleij MC, Clark JC, Baron JC, Aigbirhio FI. Rapid preparation of (11C)flumazenil: Captive solvent synthesis combined with purification by analytical sized columns. *J Labelled Comp Radiopharm*. 2007;50(1). doi:10.1002/jlcr.1152
2. Geeraerts T, Coles JP, Aigbirhio FI, et al. Validation of reference tissue modelling for [11C]flumazenil positron emission tomography following head injury. *Ann Nucl Med*. 2011;25(6):396-405. doi:10.1007/S12149-011-0480-4/FIGURES/7
3. Friston KJ, Holmes AP, Poline JB, et al. Analysis of fMRI time-series revisited. *Neuroimage*. 1995;2(1):45-53. doi:10.1006/nimg.1995.1007
4. Wu Y, Carson RE. Noise reduction in the simplified reference tissue model for neuroreceptor functional imaging. *J Cereb Blood Flow Metab*. 2002;22(12):1440-1452. doi:10.1097/01.WCB.0000033967.83623.34
5. Tustison NJ, Avants BB, Cook PA, et al. N4ITK: Improved N3 bias correction. *IEEE Trans Med Imaging*. 2010;29(6):1310-1320. doi:10.1109/TMI.2010.2046908
6. Avants B, Tustison NJ, Song G. Advanced Normalization Tools: V1.0. *Insight J*. 2009;2:618. doi:10.54294/uvnhin
7. Isensee F, Schell M, Pflueger I, et al. Automated brain extraction of multisequence MRI using artificial neural networks. *Hum Brain Mapp*. 2019;40(17):4952-4964. doi:10.1002/hbm.24750
8. Yeh FC. Shape analysis of the human association pathways. *Neuroimage*. 2020;223. doi:10.1016/j.neuroimage.2020.117329
9. Yeh FC. Population-based tract-to-region connectome of the human brain and its hierarchical topology. *Nature Communications 2022 13:1*. 2022;13(1):1-13. doi:10.1038/s41467-022-32595-4
10. Yeh FC, Verstynen TD, Wang Y, Fernández-Miranda JC, Tseng WYI. Deterministic diffusion fiber tracking improved by quantitative anisotropy. *PLoS One*. 2013;8(11). doi:10.1371/journal.pone.0080713

© 2024 Woodrow RE et al. *JAMA Network Open.*
