# Supplementary material for: Outcomes and Mechanisms Associated With Selective Thalamic Neuronal Loss in Chronic Traumatic Brain Injury
Source: JAMA Netw Open. 2024 Aug 6;7(8):e2426141. doi: 10.1001/jamanetworkopen.2024.26141 (PMC11304117; doi:10.1001/jamanetworkopen.2024.26141)
Supplement: Supplement 2. — Data Sharing Statement [file jamanetwopen-e2426141-s002.pdf]

Data Sharing Statement

Woodrow. Outcomes and Mechanisms Associated With Selective Thalamic Neuronal Loss in Chronic Traumatic Brain Injury. *JAMA Netw Open*. Published August 06, 2024. doi:10.1001/jamanetworkopen.2024.26141

Data
Data available: No
